# Single aortic clamping during coronary artery bypass surgery reduces cerebral embolism and improves neurocognitive outcomes

**DOI:** 10.1186/1749-8090-8-S1-P54

**Published:** 2013-09-11

**Authors:** M Borojevic, H Gašparović, B Malojčić, B Biočina, T Gašparović

**Affiliations:** 1 Department of Cardiac Surgery, University Hospital Rebro Zagreb, Zagreb, Croatia; 2 Department of Neurology, University Hospital Rebro Zagreb, Zagreb, Croatia; 3 Department of Cardiology, University Hospital Rebro Zagreb, Zagreb, Croatia

## Background

Aortic manipulation is responsible for the release of variable amounts of embolic material into the circulation, thereby enhancing the probability of neurologic injury. In the present study, transcranial Doppler (TCD) imaging was used to quantify the embolic load of patients undergoing CABG in relation to different aortic clamping strategies.

## Methods

59 patients undergoing elective CABG were prospectively evaluated. Two groups of patients were formed, based upon the aortic clamping strategy utilized to achieve myocardial revascularization. The single aortic clamp group (SC) whom CABG was performed using a single period of aortic clamping, whereas 22 patients in the multiple aortic clamp group (MC) group had their aorta side-clamped for the construction of proximal anastomoses. The neurocognitive data acquisitions were performed preoperatively (PRE), on postoperative day 7 (POD7) and at 4-month follow-up (F-U).

## Results

The preoperative neurocognitive results were comparable between the groups (P>0.05 for all comparisons). The majority of POD 7 neurocognitive evaluations were significantly depressed in comparison to preoperative results in both groups (P<0.05 for multiple comparisons). The magnitude of this cognitive depression, however, was significantly greater in the MC group (P<0.05 for multiple comparisons). Preoperative levels of neurocognition were restored at 4 month F-U in the SC group in all tests except the Rey AVLT. A trend towards improvements in neurocognitive performances at F-U vs POD7 was also observed in the MC group. In contrast to the SC group, however, residual attention, motor skill and memory deficits were documented with all of the tests utilized to quantify neurocognitive outcomes.

## Conclusion

The embolic burden associated with single aortic clamping for myocardial revascularization is lower than that seen with the conventional multiple clamping strategy.

